# Challenges in valuing and paying for combination regimens in oncology: reporting the perspectives of a multi‐stakeholder, international workshop

**DOI:** 10.1186/s12913-021-06425-0

**Published:** 2021-05-03

**Authors:** Nicholas. R Latimer, Daniel Pollard, Adrian Towse, Chris Henshall, Lloyd Sansom, Robyn L Ward, Andrew Bruce, Carla Deakin

**Affiliations:** 1 School of Health and Related Research, University of Sheffield, Regent Court, 30 Regent Street, S1 4DA Sheffield, UK; 2 Office of Health Economics, London, UK; 3 School of Pharmacy and Medical Sciences, University of South Australia, Adelaide, Australia; 4 Faculty of Medicine and Health, University of Sydney, Sydney, Australia; 5 Amgen, Sydney, Australia; 6 National Institute for Health and Care Excellence, Manchester, UK

**Keywords:** Cancer, Combination therapy, Cost‐effectiveness, Costs, Economic evaluation, Value‐based pricing, Pricing, Reimbursement

## Abstract

**Background:**

It is increasingly common for two or more treatments for cancer to be combined as a single regimen. Determining value and appropriate payment for such regimens can be challenging. This study discusses these challenges, and possible solutions.

**Methods:**

Stakeholders from around the world attended a 2-day workshop, supported by a background paper. This study captures key outcomes from the discussion, but is not a consensus statement.

**Results:**

Workshop attendees agreed that combining on-patent treatments can result in affordability and value for money challenges that delay or deny patient access to clinically effective treatments in many health systems. Options for addressing these challenges include: (i) Increasing the value of combination therapies through improved clinical development; (ii) Willingness to pay more for combinations than for single drugs offering similar benefit, or; (iii) Aligning the cost of constituent therapies with their value within a regimen. Workshop attendees felt that (i) and (iii) merited further discussion, whereas (ii) was unlikely to be justifiable. Views differed on the feasibility of (i). Key to (iii) would be systems allowing different prices to apply to different uses of a drug.

**Conclusions:**

Common ground was identified on immediate actions to improve access to combination regimens. These include an exploration of the legal challenges associated with price negotiations, and ensuring that pricing systems can support implementation of negotiated prices for specific uses. Improvements to clinical development and trial design should be pursued in the medium and longer term.

## Background

Cancers usually arise through the accumulation of multiple genetic events or genomic alterations and often develop resistance to single drug treatments [1, 2]. For this reason, combination regimens have been the mainstay of treatment for many cancers. Historically, these regimens tended to combine newer on-patent medicines with low-cost off-patent medicines. Recently it has become common for two or more on-patent treatments to be combined. This reflects an increasingly large drug development pipeline and a desire to make new treatments available to patients quickly. Often several new treatments are introduced in a short space of time, and frequently these are combined into a single treatment regimen. The end result is expensive treatments, leading to significant affordability challenges for many payers. Furthermore, combination regimens are often found not to represent good value for money by health technology assessment (HTA) agencies and pricing and reimbursement bodies [2]. For these reasons, patient access to effective novel combination therapies for cancer is restricted or denied in many health systems.

In this paper we explore the challenges in valuing and paying for combination regimens in cancer, drawing on discussion at an international workshop on this topic, held in Sydney in November 2019. Our focus, and the focus of the workshop, is on combination therapies because of their preponderance in cancer, and because they typically result in more expensive regimens and more complex pricing and valuation considerations than monotherapies. There has been an academic discussion of the topic in the literature, [1–5] but, interactions with payers, patient organisations and industry indicate that they all feel that action is required. Our paper reports on the views of senior representatives of these and other stakeholders from around the world attending the Sydney Workshop on the challenges and – critically – how to address them. We identify where there is agreement on how to proceed and on where further discussion is needed. In this paper, first we describe the valuation and payment challenges raised by combination therapies in cancer. Next, we summarise the set-up of the Sydney Workshop. We then outline the options for addressing these challenges discussed at the Workshop. Finally, by way of discussion, we suggest a series of actions for addressing the challenges, before offering concluding remarks. Throughout, we attempt to capture what emerged from extended discussions at the Workshop, summarising material from the much longer meeting report [6] in order to make it more accessible and likely to impact future policy discussion. This paper is not a consensus statement from those present at the Workshop or the organisations they are associated with. It is a contribution to the debate, aiming to stimulate discussion (and hopefully agreement and action) within and between the various stakeholder groups, nationally and internationally.

### Challenges associated with combination drug regimens in cancer

In this section we highlight the problems associated with the valuation and pricing of combination therapies using two hypothetical scenarios, and one real example. Then we consider whether the challenges differ depending upon the HTA and pricing systems in place.

#### Hypothetical scenarios

Combination therapies are usually developed in one of two ways: two or more existing treatments might be combined, or one new treatment might be added to existing therapy.

First, consider two drugs that already exist as monotherapies. Both bring a value of ‘1’ when given as monotherapies, and are priced to value, so both have the same price, say ‘1’. Now assume that combining the two drugs provides a value of 1.5, at a cost of 2 (1 + 1). The combination regimen adds value, but whilst the total cost of therapy has doubled, value has not. Hence, the combination would be unlikely to be considered good value for money.

Second, consider a case where one drug exists as monotherapy, and an add-on treatment is developed. The existing monotherapy must be given in every additional month lived, and is priced so that providing each extra month of life is just supported by the added value that is delivered. When combined with the existing therapy, the new add-on treatment results in patients living an extra 12 months, but, as a result, requires 12 months more treatment with the existing therapy. Because it is already priced at the boundary of what the system is prepared to pay for each additional month of survival, there is little or no headroom left for any additional costs associated with the new add-on therapy. Hence, the combination would not be considered good value for money – in some circumstances, even if the add-on therapy was provided at zero price. This would be true, irrespective of the number of additional months survival combination therapy produced.

#### Pertuzumab – a real world example

 In 2013, pertuzumab in combination with trastuzumab and docetaxel was appraised by the National Institute for Health and Care Excellence (NICE), for adults with human epidermal growth factor 2 (HER2)-positive metastatic or locally recurrent unresectable breast cancer [7, 8]. Pertuzumab was an add-on treatment, and trastuzumab and docetaxel represented backbone therapy. It is important to note that the initial NICE appraisal of pertuzumab was undertaken before the final analysis of the pivotal clinical trial was available [7]. At this time, data on overall survival (OS) were limited, with median survival not yet reached in the pertuzumab arm of the pivotal study [7]. NICE guidance on pertuzumab was reviewed in 2018, at which point stronger evidence on OS was available [9]. It is worth noting that in the real world, HTA and payer bodies are often required to make initial assessments of treatments in the face of substantial uncertainty around key endpoints, so this example is not unusual.

During its initial appraisal, NICE’s Appraisal Committee concluded that adding pertuzumab to trastuzumab and docetaxel provided progression-free survival (PFS) gains of approximately 6 months [7, 8]. In the absence of confirmatory data, the OS advantage associated with pertuzumab was considered to be highly uncertain, and in one scenario analysis considered by the Appraisal Committee it was assumed that there would be no additional post-progression survival gain attributed to the addition of pertuzumab – thus, the 6 month PFS gain would lead to a 6 month OS gain. According to calculations by NICE’s Decision Support Unit (DSU), the additional 6 months cost of remaining in PFS – comprising of backbone drug and administration costs and supportive care costs – was £13,627, even if pertuzumab had zero price [3]. The quality adjusted life year (QALY) gain of 6 months spent in PFS was 0.39, using a utility score of 0.79 [3]. Therefore, the incremental cost per QALY gained from the 6 month PFS gain, with zero price attributed to pertuzumab and no additional OS gain, was £34,712 (£13,627/0.39). Given NICE typically considers new treatments cost-effective if they provide one QALY for an incremental cost of less than £20,000 to £30,000, [10] pertuzumab would not normally be considered cost-effective even if it had zero price. The Pharmaceutical Benefits Advisory Committee of Australia also conducted an appraisal of pertuzumab in this indication, used similar analyses, and drew similar conclusions [11]. Whilst this analysis only constituted one scenario considered by the NICE Appraisal Committee, and, as noted, this appraisal was reviewed in 2018 when more OS evidence was available, this pertuzumab example illustrates a case where a new add-on therapy could be considered “not cost-effective even at zero price”.

#### Do the challenges depend on HTA and pricing systems?

Two approaches are commonly adopted by HTA agencies when assessing the value of treatments: a “therapeutic added value” approach with outcomes expressed in clinical terms; or a “QALY approach”, where clinical outcomes are translated using utilities into QALYs. The former is an approach used in France and Germany, whereas the latter is used in the UK, Sweden, Canada and Australia.

Whichever approach is taken, when appraising combination therapies HTA agencies and payers must address the following issues:

1. Do the combination of drugs produce outcomes that justify their overall cost (or, given the expected outcomes, what overall cost would be appropriate)?
2. Given the expected outcomes, can an acceptable price be negotiated for the drugs involved?

Irrespective of whether a therapeutic added value or a QALY approach is taken, it may happen that the combination regimen is more effective than the backbone therapy alone, but the producer and the payer/HTA agency cannot agree upon a satisfactory price. It may even be the case that no non-zero price exists for the add-on therapy that would be considered to represent good value for money.

Issues such as “not cost-effective at zero price” become most apparent in systems that explicitly estimate cost-effectiveness, but issues of access, affordability, and valuation of combination regimens also exist in countries where HTA focuses on added clinical benefit [2]. The HTA authority determines the therapeutic added value of the new combination compared to the existing backbone. Based upon this, the pricing authority considers the total cost of the combination regimen, comprising of the cost of the backbone therapy (which will have an existing price) and the cost of the new add-on treatment. Where the existing price of the backbone component of the regimen is high, the headroom for an acceptable price for the add-on therapy may be low. This could create access issues, if the price that the authority is prepared to pay for the add-on therapy is unacceptable to the producer of that therapy. In addition, issues around valuation and value attribution are highly likely to arise, if the relative price split between the backbone and add-on therapies is predicated on the pre-existing price of the backbone therapy and perceived to be inequitable.

Jurisdictions around the world also differ with respect to pricing systems. In “price taking” systems, the price initially set by the manufacturer is taken into account by the HTA agency or payer in their assessment of whether the treatment represents good value for money. In “price setting” systems, the payer initially determines the price at which they are willing to permit reimbursement, based upon their assessment of the value that the new treatment provides.

In practice, most systems involve some element of price negotiation, implicit or explicit. However, in the context of combination regimens, manufacturers only have the power to amend the price of a portion of the regimen, if constituent parts are provided by different companies. Thus, price negotiations may be less straightforward and more restricted in the context of combination regimens. Further, in a system that is price taking in nature, there would appear to be little opportunity for renegotiation of a pre-existing price of an existing backbone therapy during an assessment of a novel add-on treatment. In contrast, in theory at least, a price-setting payer would appear more able to take the initiative and re-set the price that they are willing to pay for a backbone therapy during an assessment of a novel combination regimen. The practicalities of changing the price of a backbone therapy when used in combination are likely to be country-specific and may depend, for example, on existing pricing and reimbursement legislation, the responsibilities of individual national agencies and mechanisms currently available to achieve price changes.

## Methods

### The Sydney International Workshop

 The Workshop was convened by Bellberry, a not-for-profit organisation that promotes and improves the welfare of research participants and the quality of research [12]. The Workshop was conducted over two days and attended by fifty-three people from patient organisations, regulators, HTA/payer bodies, universities (ethicists, statisticians and health economists), and life sciences companies from Australasia, Asia, Europe, and North America. The programme included presentations from various attendees, and plenary and break-out group discussions. Attendees received in advance a detailed agenda for the meeting, a background paper, [13] and copies of five relevant publications and reports [1–3, 14, 15]. The development of these materials was overseen by a Scientific Committee (see the meeting report for further details on attendees [6]).

The pre-read documents provided to attendees included a literature review of options to address the valuation and payment challenges presented by combination regimens in cancer [13]. In addition, prior to the meeting, attendees were asked for potential solutions/ways forward, and whether they were engaged in, or aware of, any current work in the area. Further ideas emerged as discussion as the meeting progressed.

Participants agreed that attendees should be free to report anything said in the discussions, but not which participants said it. They also agreed that the background paper and a full meeting report should be publicly available, [6, 13] but that the slides presented should be available only to participants. These arrangements were designed to protect potentially sensitive information and to promote free and open discussion by ensuring the confidentiality of individuals’ contributions.

## Results

### Challenges - summary

 Attendees at the Sydney Workshop agreed that the challenges associated with valuing and paying for combination therapies in oncology were truly international. Although challenges manifest in different ways between systems, affordability, value for money and value attribution were consistently challenging issues that can impact patient access.

Whilst attendees agreed that the challenges outlined above are common and important, they were keen to clarify that the challenges only arise when more than one *on-patent* treatments are combined, and when *different* manufacturers produce the constituent parts of a combination. It was, however, noted that a different challenge to patient access to new combination therapies can arise where old drugs are repurposed and found to be clinically effective in a new low-cost combination use. Often these regimens lack a manufacturer sponsor to take them through regulatory and HTA processes. This is tangential to the challenges associated with providing access to new high cost combination regimens, but is an important issue that needs addressing [16–18].

### Options for addressing the challenges presented by combination regimens

**Fig. 1:**
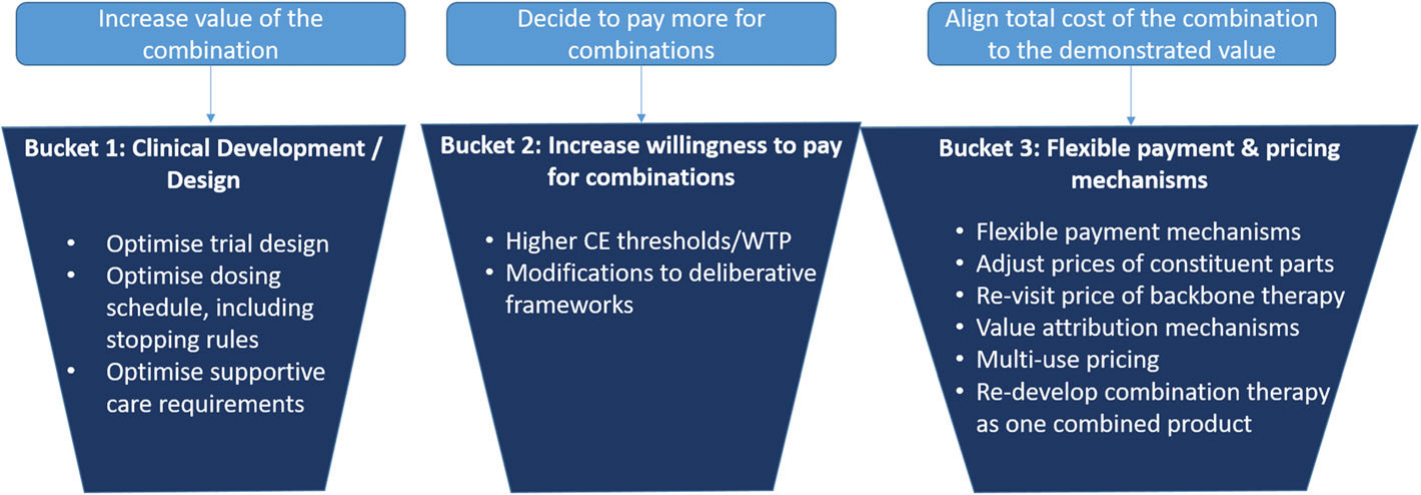
Three Buckets – options to address the challenges associated with valuing and paying for combination therapies in cancer. CE: Cost-effectiveness, WTP: Willingness to Pay

Attendees categorised potential solutions into three ‘buckets’ (Fig. 1). The general challenge amounted to the cost of combination regimens often being too high, given their perceived and/or assessed value when price is determined. The three buckets of solutions involved:

1. Increasing the value of combinations, through improved clinical development and trial design to optimise clinical regimens.
2. Being willing to pay more for combinations (over and above single treatments offering comparable value).
3. Aligning the total cost of the combination to the demonstrated value, by using flexible payment and pricing mechanisms to adjust the prices of individual constituent medicines.

The remainder of this section describes each of these options as they were discussed at the Workshop.

### Bucket 1: Increase the value of the combination – clinical development and trial design to optimise clinical regimens

Attendees considered it may be possible to increase the benefits associated with combination regimens whilst potentially reducing their costs through optimising treatment regimens. This idea was not identified in the literature – it was suggested by Workshop attendees.

Optimising treatment regimens may involve altered dosing schedules, treatment durations and supportive care requirements. Reduced toxicity, improved quality of life, and lower costs could result, increasing the likelihood that treatments would represent good value for money. Discussion focused primarily on the use of stopping rules. It was noted that sometimes submissions to HTA agencies attempt to model these, but evidence on their impact on effectiveness is usually lacking – making it difficult for HTA agencies and payers to incorporate stopping rules into their decision making. It was felt that, if treatment regimen alterations such as stopping rules are deemed clinically valid and potentially cost-saving, evidence on their effectiveness should be collected.

Targeting combination regimens specifically at patient groups who are most likely to benefit from them was also identified as an approach that could increase the value of new treatments. Whilst valuable, this is difficult, because it is likely to require development of diagnostic tests and/or improved monitoring of patients to identify responders (or those most likely to respond). In addition, attendees agreed that it was important to ensure that all relevant outcome measures were collected in trials, including patient relevant measures, to ensure the true value of therapies can be demonstrated.

Attendees supported increased use of adaptive trial designs and platform trials, which could allow altered dosing regimens, stopping rules or diagnostic tests to be incorporated within ongoing trials [19–22]. Attendees supported the use of combined scientific advice processes – whereby HTA agencies, payers and regulators provide joint advice to manufacturers on trial design and clinical development programmes. This could identify at an early stage of clinical development cases where investigation of alternative treatment regimens could be particularly worthwhile – allowing useful data to be collected. Also, there was agreement that pharmaceutical companies and HTA agencies/payers should work to make more use of post-launch randomised and observational studies to provide information on alternative treatment regimens.

### Bucket 2: Pay more for combinations

Attendees recognised that HTA processes could be amended to increase willingness to pay for combination therapies for cancer. Most HTA agencies have flexible decision-making criteria, such that recommendations are not based solely on whether a new treatment provides value for money according to rigidly specified thresholds or criteria. For example, ‘therapeutic added value’ systems usually compare the benefits and costs of a new treatment and those of other treatments for the same condition, with no explicit reference to a willingness to pay threshold. In systems that do specify thresholds, these are often flexible – NICE specifies a threshold range, which differs for end of life treatments and technologies defined as “highly specialised” [10, 23]. And in Sweden, different thresholds are used for different disease areas [24]. In general, it is not unusual for orphan drugs to be given special consideration by HTA and payer bodies [25]. Given this flexibility, ‘combination therapies’ could be used as a modifier in the decision-making or value assessment framework, permitting higher prices. This approach has been suggested in the literature [2].

Attendees generally agreed that there are benefits to having flexible cost-effectiveness thresholds, broader value frameworks and flexible deliberative processes incorporated into HTA. Attendees agreed on the importance of HTA processes capturing the full value of all therapies assessed. However, they considered that special provisions could only be made for combination therapies for cancer with an evidence-based justification. Evidence that society values combination therapies more highly than other treatments, including monotherapies for cancer, would be needed. Workshop attendees were not aware of any such evidence, and thus could see no grounds for such special provisions for combination therapies. It was recognised that if willingness-to-pay was increased for combination therapies and this was not consistent with societal preferences, this could come at the expense of other treatments offering more health gain, resulting potentially in net societal losses.

### Bucket 3: Aligning the total cost of the combination to the demonstrated value – flexible payment and pricing mechanisms

Workshop attendees generally agreed that when existing monotherapies are combined and produce value that is not proportional to their combined cost, it would be appropriate for constituent prices to be re-visited and negotiated. Similarly, when new add-on treatments are combined with an existing backbone therapy and provide clinical benefit, it is appropriate for the price of the backbone therapy to be re-visited in its new use. This has been suggested in the literature, [2, 4] but raises issues of implementation, value attribution, and legal challenge, when the constituent therapies are made by different manufacturers. These issues were discussed at length at the Sydney Workshop, as reflected below. Another suggestion in the literature [4] - that combination regimes could be re-developed as single products - was also discussed but felt to be impractical. Similarly, attendees expressed a concern that without progress on ways to revisit and adjust prices of constituent therapies, manufacturers of new add-on treatments might decide to develop their own “me-too” versions of backbone therapies (over which they would have control of price), representing an inefficient use of valuable drug development resources.

#### Bucket 3: Implementation

Attendees recognised that if the prices of existing treatments are to be re-visited as part of a combination regimen price negotiation, a key consideration is whether the price is changed for all uses of the treatments (i.e. for their use as monotherapies, and/or their use in other disease areas), or only for their use as part of the combination therapy being appraised. To limit dis-incentives to price negotiation, attendees suggested multi-use pricing is likely to be required – allowing prices to differ for a treatment depending upon the disease area it is being used in, and/or depending on whether it is being used as monotherapy or as part of combination therapy [2, 14, 15]. Price discounts or budget caps could also be used to achieve price reductions for specified uses.

Pricing systems to allow multi-use pricing may be complex and costly to run and may need to be taken into account in assessments of value for money. Any multi-use pricing mechanism would ideally be based on good data on the use of cancer treatments, including clinical indication, therapy line, type of combination, dosing and treatment duration. In some countries, potentially appropriate data are already collected (e.g. the Systemic Anti-Cancer Therapy dataset in England [26]). Alternatively, reasonable assumptions about differential use of treatments would need to be agreed, based on epidemiological data and whatever health system data are available. Or, this information combined with a value assessment for each use of a product could be used to calculate an appropriate weighted average price across all uses of a product. Attendees noted that some countries lack sufficient flexibility to make this work.

Workshop attendees felt that the exact method used to implement price adjustments for constituent parts of a combination regimen was relatively unimportant – whether through multi-use pricing, discounts, budget caps, or combinations of these. However, having a system in place that could support such methods was crucially important – without this, price negotiation could not achieve a solution to the challenges raised by combination regimens. Attendees agreed that HTA agencies and payers should communicate clearly with manufacturers types of flexible pricing model acceptable to achieve price reductions for combination uses. In some countries, suitable pricing mechanisms and data collection systems may need to be developed.

Workshop attendees recognised a further barrier to re-visiting the prices of constituent parts of combination regimens concerns incentives. If a backbone therapy is in use and produced by one company, and a new add-on therapy is developed by another company, has the manufacturer of the backbone therapy sufficient incentive to enter into price negotiations with the producer of the add-on? [4]. In principle, patient access to the combination therapy could increase backbone therapy sales. If, however, negotiations are likely to reduce the price of the backbone therapy in the use under appraisal and in its other uses, the company may see limited gain, and could incur losses, from negotiating. The length of time remaining on the patent of a backbone therapy may also influence the willingness of a producer (and a payer) to negotiate, as might the producer’s own drug development pipeline. Multi-use pricing could alleviate some of the dis-incentives to negotiation, but attendees also suggested that appraisals of combination regimens that raise issues around the value for money of backbone therapies in the new use should trigger the re-assessment of (and possible disinvestment in) the use of backbone therapies in their existing uses. This could act as an incentive for manufacturers to negotiate.

#### Bucket 3: Value attribution

If the prices of the constituent parts of combination regimens are to be negotiated, consideration must be given to *how* prices (or values) of these parts should be determined, and *who* they should be determined by. This was of particular concern to a number of attendees.

Options for ‘how’ value could be attributed between constituent parts of a combination regimen have been discussed in the literature [2, 4, 13]. Simple options exist, for example, splitting the revenue equally. A formal quantitative value attribution framework could be used, where value is based upon the estimated benefit that each constituent part contributes to the combination – though this may not be straightforward to calculate. Attendees felt that research into value attribution frameworks for combination regimens would be useful, and should involve multiple stakeholders (including HTA agencies and academia) to increase credibility.

Attendees differed as to ‘who’ should be responsible for attributing value to constituent parts of combination therapy. Some attendees felt this was the responsibility of the pharmaceutical companies – several HTA representatives felt strongly that HTA agencies and payers are responsible for assessing the value of overall treatment packages, not individual constituent parts of combination regimens. Other attendees felt that value attribution was a natural role for HTA agencies and/or payers, because their remit was to value healthcare interventions. Even if an HTA agency did not feel it appropriate to attribute value to constituent parts of a therapy, there might be an important role for it as a broker of discussions between companies. Whoever attributes value, it was recognised that deliberative processes would be required. It is unlikely that prices could be set solely using quantitative methods. There was agreement that value attribution should be addressed early in the HTA process – ideally before reimbursement submission to HTA agencies or payers – to avoid delays in the appraisal process, which would delay patient access.

#### Bucket 3: Legal challenges

Many attendees were concerned about the legal challenges that price negotiations between companies present. Legal experts explained the issues, including competition law around collusion. Some attendees suggested that participation of a third party – an HTA agency or payer – may help, echoing the literature [2]. Attendees were also told about a platform designed to enable companies to trade, without meeting, under the supervision of HTA agencies. However, legal experts explained that the involvement of HTA agencies and payers in price negotiations may not solve the legal problems, and in some circumstances may raise additional issues.

Attendees recognised that the legal issues are critical, and may dictate whether price negotiations offer a practical solution to providing affordable access to effective combination therapies. Attendees strongly agreed there was an urgent need for pharmaceutical companies and HTA agencies/payers to explore the legalities of price negotiations between companies (with or without the involvement of HTA agencies and payers) recognising that what is permitted may vary by jurisdiction, and may require amended legislation.

## Discussion

Attendees from all around the world agreed that combination therapies in cancer present important problems for affordability, value for money, and patient access. There was substantial support for actions to improve patient access to clinically effective high-cost combination therapies. These actions are listed in Table 1, highlighting which stakeholders have responsibility for taking next steps. More discussion is required within specific jurisdictions to agree action plans and allocate actions to different stakeholders.

**Table 1:** Suggested actions, based upon discussion heard at the Sydney Workshop

Bucket	Action	Description
1	1	Thought should be given to the treatment regimens tested in clinical trials, particularly with respect to stopping rules and targeting treatments to those patients most likely to benefit. Adaptive and platform trials may be useful practically and from an ethical perspective. Combined scientific advice processes should be considered to enable this, including all relevant stakeholders (e.g. different **HTA agencies, payers, regulators, patients, clinicians, ethicists, academics**).
	2	Research is required to identify the most patient-relevant outcome measures to be included in clinical trials. This requires input from **all stakeholders.**
	3	**Manufacturers and HTA agencies/payers** should work to make more use of post-launch studies and real world data to provide information on alternative treatment regimens including the use of stopping rules.
2	4	**HTA agencies** should ensure that assessments of combination therapies (like assessments of any treatment) capture the full value of the therapy. There is not currently a case for altering HTA decision rules or deliberative frameworks specifically for combination therapies.
3	5	When combination therapies are assessed by **HTA agencies/payers** and issues arise regarding the value for money of existing backbone therapies, this should trigger a re-assessment of the backbone therapy by the **HTA agency/payer**.
	6	**Manufacturers** should not develop combination therapies as single products (except where there are clinical benefits from doing so). This is inefficient and often impossible. Similarly, all stakeholders should work to remove incentives for manufacturers to develop their own “me too” versions of backbone therapies simply to achieve value for an effective add-on technology.
	7	**Manufacturers** should be prepared to revisit the price of backbone therapies (in respect of their use within the combination) when add-on therapies are combined and provide clinical benefit, with a view to ensuring that the price of the combination therapy is commensurate with its value whilst also allowing prices for constituent parts that are acceptable to their manufacturer. Similarly when existing monotherapies are combined.
	8	It is important for **HTA agencies and payers** to communicate clearly with **manufacturers** what type of multi-use flexible pricing models are implementable to achieve price adjustments in their jurisdiction.
	9	**Health systems** need to have in place appropriate systems to collect data on the actual or likely use of cancer treatments, if flexible pricing and payment mechanisms are to be used.
	10	It is important for **all stakeholders** to consider how to incentivise companies (particularly manufacturers of backbone therapies) to participate in price negotiations.
	11	There is a range of views on who (**manufacturer, HTA agency, payer**) should be responsible for attributing value to the constituent parts of a combination therapy. This needs to be discussed and agreed within specific jurisdictions.
	12	Research – involving **all stakeholders** – into how value could be attributed between constituent parts of a combination therapy would be valuable.
	13	There is an urgent need for **manufacturers, payers and HTA agencies** to explore the legalities of price negotiations between companies in different jurisdictions around the world. **HTA agencies/payers** have – at least – an important facilitation role in enabling price negotiation.
	14	It is important for **pharmaceutical companies, regulators, HTA agencies and payers**, to further consider how to provide patient access to new low-cost combination therapies. These typically involve repurposed drugs that are found to be newly clinically effective in combination uses. As they are off-patent, there is no manufacturer sponsor to take them through the regulatory and HTA processes. This is not an issue relating directly to high cost combination therapies, but one that is potentially important to improving the quality of cancer care through new uses for off-patent medicines.

It is important to note that whilst the Sydney Workshop included many stakeholders from several countries around the world, it did not specifically include attendees who would provide an international, rather than nation-specific, perspective. This may be important, because pharmaceutical companies are typically global, and operate within global investment markets, and therefore their decision-making needs to be understood from this perspective. The interaction between national-specific HTA agencies and payers with global pharmaceutical companies operating in international markets may be an important consideration in future thinking to address the challenges associated with combination therapies for cancer.

Whilst implementing systems that allow pricing negotiations to take place represents an important short-term step towards allowing combination therapies for cancer to represent good value for money, we believe that improved clinical development programmes are worthy of further investigation. In further research it would be useful to investigate the potential impact on value calculations of improved benefits associated with previously appraised combination therapies to examine whether, for example, shorter overall treatment regimens, or reduced toxicity through altered dosing schedules could lead to different value-for-money conclusions.

## Conclusions

There is an urgent need for pharmaceutical companies, HTA agencies and payers to work together. Improving clinical development programmes is essential but will take time to achieve. More immediately, legally permissible approaches to price negotiations between companies (with or without HTA agencies) need to be identified or developed in jurisdictions around the world, and pricing systems need to be made flexible enough to implement multi-use prices in some form.

## Data Availability

Not applicable – our manuscript does not contain any data.

## Abbreviations

HTA: Health Technology Assessment
NICE: National Institute for Health and Care Excellence
HER2: Human Epidermal Growth Factor 2
OS: Overall Survival
PFS: Progression-free Survival
DSU: Decision Support Unit
QALY: Quality Adjusted Life Year
WTP: Willingness to Pay
CE: Cost-effectiveness

## References

[1] Humphrey RW, Brockway-Lunardi LM, Bonk DT, Dohoney KM, Doroshow JH, Meech SJ (2011). Opportunities and Challenges in the Development of Experimental Drug Combinations for Cancer. J Natl Cancer Inst.

[2] Danko D, Blay JY, Garrison LP. Challenges in the value assessment, pricing and funding of targeted combination therapies in oncology. Health Policy. 2019;123:12:1230–6. 10.1016/j.healthpol.2019.07.009 31337514

[3] Davis S. Assessing technologies that are not cost-effective at zero price. Report by the Decision Support Unit, July 2014. Available from http://www.nicedsu.org.uk. Accessed 23 Mar 21.

[4] Persson U, Norlin JM. Multi-indication and Combination Pricing and Reimbursement of Pharmaceuticals: Opportunities for Improved Health Care through Faster Uptake of New Innovations. Appl Health Econ Health Policy. 2018;16:2:157–65. 10.1007/s40258-018-0377-7 29470774

[5] Latimer NR, Towse A, Henshall C. Not cost-effective at zero price: valuing and paying for combination therapies in cancer. Expert Rev Pharmacoecon Outcomes Res., 2021.;1–4. 10.1080/14737167.2021.1879644. Online ahead of print. 10.1080/14737167.2021.1879644 33472440

[6] Latimer N, Pollard D, Towse A, Henshall C. Challenges in valuing and paying for combination regimens in oncology. Report of an international workshop convened by Bellberry. 2020. Available from: https://bellberry.com.au/wp-content/uploads/Meeting-report-final-draft-May-2020.pdf,. Accessed 08 Mar 21. 10.1186/s12913-021-06425-0 PMC8091555 33941174

[7] The National Institute for Health and Care Excellence (2013). Breast cancer (HER2 positive, metastatic) - pertuzumab (with trastuzumab and docetaxel): appraisal consultation document.

[8] Fleeman N, Bagust A, Beale S, Dwan K, Dickson R, Proudlove C, Dundar Y, O’Reilly S. Pertuzumab in combination with trastuzumab and docetaxel for the treatment of HER2 positive metastatic or locally recurrent unresectable breast cancer. LRiG, The University of Liverpool; 2013. Available from: https://www.nice.org.uk/guidance/ta509/documents/breast-cancer-her2-positive-metastatic-pertuzumab-with-trastuzumab-and-docetaxel-evaluation-report2. Accessed 23 Mar 21. 10.1007/s40273-014-0206-2 25138171

[9] The National Institute for Health and Care Excellence, Pertuzumab with trastuzumab and docetaxel for treating HER2-positive breast cancer. Technology appraisal guidance. London: The National Institute for Health and Care Excellence; 2018. Availble. from www.nice.org.uk/guidance/ta509,. Accessed 08 Mar 21. 41397071

[10] National Institute for Health and Care Excellence. Guide to the methods of technology appraisal 2013. Available from: http://www.nice.org.uk/article/pmg9/chapter/foreword,. Accessed 08 Mar 2021. 27905712

[11] PBAC Public Summary Document: PERTUZUMAB, 420 mg/14 mL injection, 1 × 14 mL vial Perjeta®, Roche Products Pty Ltd. The Pharmaceutical Benefits Scheme, Australian Government, Department of Health. 2014. Available from: http://www.pbs.gov.au/info/industry/listing/elements/pbac-meetings/psd/2014-03/pertuzumab, Accessed 08 Mar 2021.

[12] Bellberry Limited. https://bellberry.com.au/., Accessed 14 July 2020.

[13] Latimer N, Pollard D. Pre-read document 1: Challenges in valuing and paying for combination regimens in oncology, School of Health and Related Research, University of Sheffield, 6th November 2019. Available from: https://www.sheffield.ac.uk/scharr/sections/heds/discussion-papers/20_02-1.882458,. Accessed 08 Mar 2021.

[14] Towse A, Cole A, Zamora B. The Debate on Indication-Based Pricing in the US and Five Major European Countries. In: OHE Consulting Report. London: Office of Health Economics; 2018.

[15] Mestre-Ferrandiz J, Towse A, Dellamano R, Pistollato M. Seminar Briefing 18: Multi-indication Pricing: Pros, Cons and Applicability to the UK. In: Office of Health Economics: Research London: Office of Health Economics; 2015.

[16] Association of Medical Research Charities. Facilitating adoption of off-patent, repurposed medicines into NHS clinical practice. London: Association of Medical Research Charities; 2017. Available from: https://www.amrc.org.uk/blog/facilitating-adoption-of-off-patent-repurposed-medicines-into-nhs-clinical-practice,. Accessed 08 Mar 2021.

[17] Pushpakom S, Iorio F, Eyers PA, Jane Escott K, Hopper S, Wells A (2019). Drug repurposing: progress, challenges and recommendations. Nat Rev Drug Discovery.

[18] Queensland Drug Repurposing Initiative, Centre for Clinical Research. The University of Queensland, Australia. Information available from: https://clinical-research.centre.uq.edu.au/qdri. Accessed 23 Mar 2021.

[19] Pallmann P, Bedding AW, Choodari-Oskooei B, Dimairo M, Flight L, Hampson LV, et al. Adaptive designs in clinical trials: why use them, and how to run and report them. BMC Med. 2018;16:29. 10.1186/s12916-018-1017-7 PMC5830330 29490655

[20] Schiavone F, Bathia R, Letchemanan K, Masters L, Amos C, Bara A, et al. This is a platform alteration: a trial management perspective on the operational aspects of adaptive and platform and umbrella protocols. Trials;. 20;264; 2019. 10.1186/s13063-019-3216-8 PMC6540525 31138317

[21] Hague D, Townsend S, Masters L, Rauchenberger M, Van Looy N, Diaz-Montana C, et al. Changing platforms without stopping the train: experiences of data management and data management systems when adapting platform protocols by adding and closing comparisons. Trials;. 2019;20:294. 10.1186/s13063-019-3322-7 PMC6540437 31138292

[22] Morrell L, Hordern J, Brown L, Sydes MR, Amos CL, Kaplan RS (2019). Mind the gap? The platform trial as a working environment. Trials.

[23] National Institute for Health and Care Excellence. Interim Process and Methods of the Highly Specialised Technologies Programme Updated to reflect 2017 changes, 2017. Available from https://www.nice.org.uk/about/what-we-do/our-programmes/nice-guidance/nice-highly-specialised-technologies-guidance,. Accessed 08 Mar 2021. 27905709

[24] Tandvårds- och läkemedelsförmånsverket (TLV). Health Economics. 2020. https://www.tlv.se/in-english/medicines/health-economics.html,. Accessed 8 Mar 2021.

[25] Heyes A, McBride D, Pearson I, Copley-Merriman K. HTA and resimbursement considerations for rare disease in European markets: What are the implications for manufacturers? Value Health. 2018;21;S3:468; PSY184.

[26] Systemic Anti-Cancer Therapy (SACT) Dataset, National Cancer Registration and Analysis Service (NCRAS), operated by Public Health England. www.chemodataset.nhs.uk,. Accessed 8 Mar 2021.

